# Globe Intussusception Following Orbital Trauma: Case Series and Review of Literature

**DOI:** 10.3390/cmtr18040044

**Published:** 2025-10-20

**Authors:** Akruti Desai, Gautam Dendukuri, Milind Naik

**Affiliations:** 1 Ophthalmic Plastic Surgery Services, Shantilal Shanghvi Eye Institute, Mumbai 400037, India; akruti.desai@ssei.ind.in; 2 Hariram Motumal Nasta & Renu Hariram Nasta, Ophthalmic Plastic Surgery Services, LV Prasad Eye Institute, Hyderabad 500034, India; drgautamd@gmail.com

**Keywords:** intussusception, eye globe, luxation, maxillary sinus, ethmoid sinus, periorbital trauma, orbital fracture

## Abstract

The aim of this paper is to report “Globe Intussusception” as an extreme form of globe dislocation outside the orbital pyramid, and provide a literature review. A single-center, retrospective, interventional case series of three patients is presented. A review of the English-language literature from the years 1971 to 2024 was performed using the search terms “traumatic globe dislocation”, “maxillary sinus” and “ethmoid sinus”. Three cases of globe intussusception are reported. Computed tomography imaging revealed orbital fracture, and globe prolapse into the maxillary sinus with or without involvement of ethmoid sinus. This was associated with complete intussusception of the globe through the conjunctiva, giving an “empty socket” appearance. In all three cases, fracture repair along with retrieval of the eyeball from the sinus was carried out surgically. Reduction of the intussusception, and bringing the eyeball out of the conjunctival pouch was a special additional challenge in these cases. The review of 35 cases reported in world literature till date is presented. We suggest retrieval of the intussuscepted eyeball via a 360° peritomy and suture tagging of extraocular muscles to ensure safe repositioning of globe with intact extraocular muscles.

## 1. Introduction

Periorbital trauma is known to cause orbital fractures and resultant enophthalmos. However, complete traumatic luxation of the globe beyond the orbital confines is an extremely rare occurrence, with approximately 35 cases reported in the literature to date. Most of these describe displacement of the eyeball into paranasal sinuses, often following high energy trauma and orbital fractures. In this series, we add three additional cases to the existing literature and introduce a novel morphological configuration observed during surgery: “*intussusception of the globe*”, a phenomenon wherein the globe appears to telescope through the conjunctiva in a manner reminiscent of intestinal intussusception. Recognizing this unique morphological configuration provides an important conceptual advance as it can guide surgeons towards safer strategies for repositioning the eyeball with preservation of adnexal and orbital structures.

Intussusception, classically known in gastrointestinal pathology, is a known phenomenon, where telescoping of one segment of intestine into another can lead to acute obstruction [1], providing a compelling analogy for this observed ocular phenomenon in traumatic luxation. Most published reports have highlighted early repositioning of a dislocated globe [2]. We report three such cases of traumatic globe luxation into the adjacent sinuses, each exhibiting this rare finding of “intussusception of the globe”. The challenges in retrieving the globe back into orbit in this special situation are discussed.

## 2. Method

This retrospective interventional case series included three patients with traumatic globe luxation into the maxillary sinus. All patients were referred to our tertiary oculoplastic service with a presumed diagnosis of anophthalmic socket. Clinical evaluation was comprised of detailed history, ocular examination and documentation of visual acuity. Computed tomography scans of the orbit in axial, coronal and sagittal planes were performed to assess orbital fractures, globe position and optic nerve integrity. All patients underwent surgical intervention for globe repositioning and orbital floor reconstruction under general anesthesia. Post-operatively, patients were followed for 12–22 months with evaluation of globe integrity, cosmesis and visual function.

A review was designed following the PICO framework to systematically identify and analyze reported cases of traumatic globe dislocation into the paranasal sinuses. The framework components were defined as follows:

- Population (P): Patients with traumatic globe dislocation or prolapse of the eyeball into adjacent paranasal sinuses (maxillary or ethmoid), as reported in English-language literature between 1971 and 2024.
- Intervention (I): Surgical retrieval and repositioning of the globe, with or without orbital floor and wall reconstruction.
- Comparison (C): Different surgical approaches and reconstructive techniques (direct traction, transconjunctival, transmaxillary, Caldwell–Luc, or combined methods).
- Outcome (O): Anatomical globe repositioning, structural integrity, and functional visual recovery post-intervention.

A systematic search was performed using the PubMed database with the keywords “traumatic globe dislocation,” “maxillary sinus,” “ethmoid sinus,” and “bull horn injury.” Only English-language case reports and series with radiological confirmation were included. Duplicate and incomplete records were excluded. The clinical parameters extracted included patient demographics, etiology of trauma, sinus involvement, time to presentation, method of globe repositioning, material used for orbital reconstruction, and post-operative visual outcome.

Due to the rarity and heterogeneity of available reports, a descriptive narrative synthesis was performed rather than a meta-analysis. Findings from the literature were compared and contextualized with the present three-case series to summarize patient demographics, sinus involvement, operative approach, outcomes, and also highlight surgical nuances in cases of traumatic globe intussusception.

Generative artificial intelligence (GenAI) has not been used in this paper to generate text, data, or graphics, or to assist in study design, data collection, analysis, or interpretation.

### 2.1. Case 1

**Figure 1:**
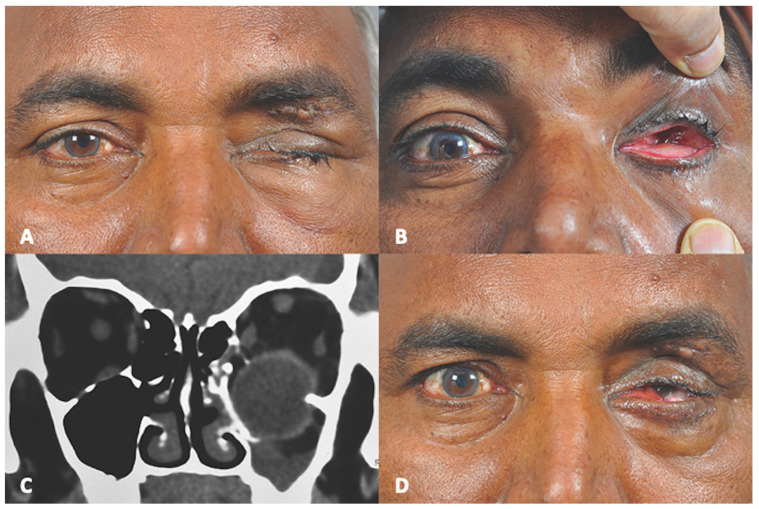
(**A**) Pre-operative Clinical Photograph showing left traumatic ptosis with sutured upper eyelid laceration. (**B**) Pre-operative Clinical Photograph showing absence of left globe in orbital cavity. (**C**) Pre-operative Computed Tomography (coronal section) showing dislocation of globe into maxillary sinus. (**D**) Post-operative Clinical Photograph showing retrieved globe in orbital space.

A 55-year-old male patient presented to us a week after a bull horn injury to the left eye. (Figure 1) His referring ophthalmologist suspected a traumatic enucleation, and referred him for the management of his socket. Best corrected visual acuity in the right eye was 20/20. Anterior segment and fundus findings were unremarkable in the right eye. Light perception was absent on the left side. Clinical examination showed no evidence of the globe on the left side. Lower eyelid fullness was noted. Computed tomography scan of the orbit showed a large floor fracture with loss of the inferomedial orbital strut. The globe was seen in the maxillary sinus. The optic nerve was seen intact in the sagittal reconstruction images. The clinical diagnosis was orbital floor fracture with globe luxation into the maxillary sinus. The patient was taken up for globe repositioning and orbital floor fracture repair under general anesthesia. Even with meticulous retraction of the conjunctiva, the eyeball could not be visualized due to the intussusception of the globe through the conjunctiva. The orbital rim was exposed, and the bony fracture opening within the floor was enlarged using Kerrison’s punch, in order to avoid rupture of the globe during its retrieval. A 360° conjunctival peritomy had to be performed within the depths of the intussuscepted conjunctiva, and the globe was visualized. While a malleable retractor kept the globe lifted into the orbit, a cryoprobe was used to prolapse the globe out through the ballooned conjunctiva. The muscles were hooked and the globe was repositioned into the orbit. Floor fracture was repaired with a silastic sheet. The patient was followed up for 15 months following the surgical repair. The left globe continued to have no perception of light. However, anatomically the globe structure remained intact.

### 2.2. Case 2

**Figure 2:**
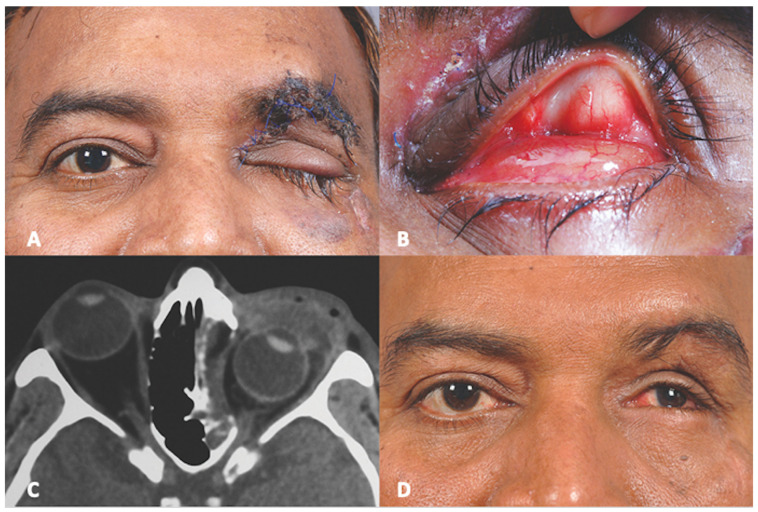
(**A**) Pre-operative Clinical Photograph showing left traumatic ptosis with sutured upper eyelid laceration (**B**) Pre-operative Clinical Photograph showing absence of left globe in orbital cavity (**C**) Pre-operative Computed Tomography (axial section) showing globe luxation (**D**) Post-operative Clinical Photograph showing globe retracted into the orbit.

**Figure 3:**
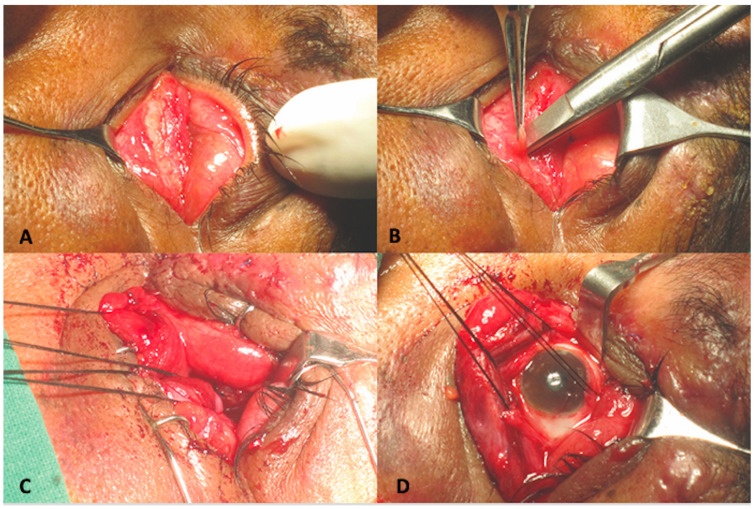
(**A**) Intra-operative photograph with no discernable globe structure in orbit (**B**) Intra-operative photograph of conjunctival peritomy in depths of intussuscepted globe (**C**) Intra-operative clinical photograph showing tagging of extraocular muscles (**D**) Intra-operative clinical photograph showing globe retrieval into the orbit through conjunctival peritomy with aid of tagged extraocular muscles.

A 45-year-old male presented to us two weeks following a road traffic accident. This patient too, was referred to us as a case of anophthalmic socket. At presentation, ocular examination revealed that right eye visual acuity was 20/30. Anterior segment and fundus findings were within normal limits in the right eye. The left eye had no light perception. Upon mechanically lifting the upper eyelid, no eyeball was visible. Computed tomography of the orbit showed a large floor and medial wall fracture with luxation of the eyeball into the maxillary sinus. (Figure 2) Similar conjunctival telescoping of the globe was noted. The patient was taken up for left orbital fracture repair and retrieval of the globe. Surgically the eyeball was first approached through a trans conjunctival incision and the globe was retrieved back into the orbit. However, conjunctival telescoping required a 360° peritomy and suture tagging of all the extraocular muscles to retrieve the globe back into its normal position, but the eyeball was intact. (Figure 3) The orbital floor and medial wall were reconstructed with a titanium mesh. The patient continued to have no light perception. Last follow up was 22 months following the surgical repair and the ocular globe integrity was maintained with acceptable cosmesis.

### 2.3. Case 3

**Figure 4:**
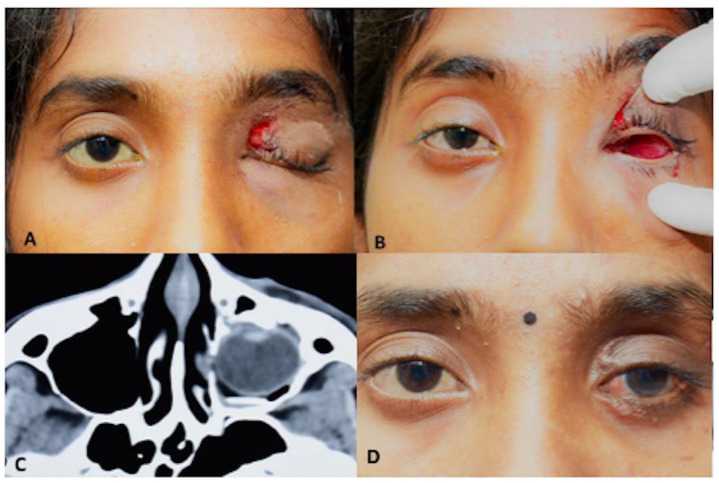
(**A**) Pre-operative Clinical Photograph showing left traumatic ptosis and upper eyelid laceration (**B**) Pre-operative Clinical Photograph showing absence of left globe in orbital cavity (**C**) Pre-operative Computed Tomography (axial section) showing dislocation of globe into maxillary sinus (**D**) Post-operative Clinical Photograph showing globe retrieved into the orbit.

**Figure 5:**
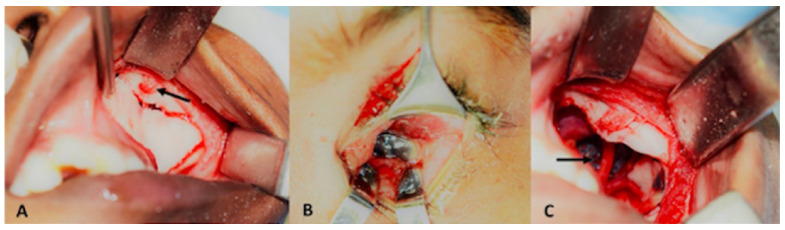
(**A**) Intra-operative photograph showing antral window below infraorbital nerve (Black arrow) (**B**) Intra-operative photograph showing globe in left orbital cavity after gentle lift from antrum (**C**) Intra-operative photograph showing intra-oral view of the floor of the orbit with titanium mesh (Black arrow).

**Figure 6:**
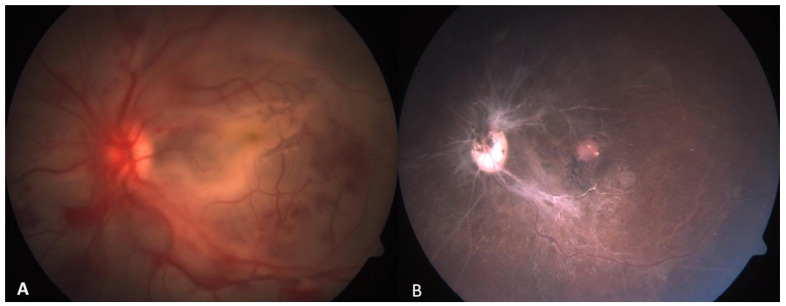
(**A**) Immediate post-operative left fundus photograph showing foveal and generalized retinal whitening with multiple pre-retinal and intra-retinal hemorrhages (**B**) Eight weeks post-operative left fundus photograph showing optic disc pallor with cupping and 360 degrees sclerosis of retinal vessels. Pigmentary changes seen at macula.

A 17-year-old woman presented with a one-day history of sudden onset painful loss of vision in the left eye following a bull horn injury. On examination, her visual acuity was 20/20 in the right eye and no perception of light in the left eye. The left orbital cavity showed no discernible globe structures. Computerized tomography of the orbit revealed orbital floor fracture with loss of inferomedial orbital strut and prolapse of the globe in the ipsilateral maxillary sinus. (Figure 4) The patient underwent surgery for left globe repositioning and fracture repair. As the eyeball had telescoped through the conjunctiva completely in the maxillary sinus, a Caldwell-Luc antrostomy was done to facilitate gentle globe reposition. The anterior wall of the maxillary sinus from the lateral end of pyriform fossa to the lateral edge of maxillary wall and from superior to insertion of canine tooth to infraorbital foramen was opened with a cutting burr and the globe gently directed to the orbit. A 360° conjunctival peritomy was done and all four recti tagged and the eyeball was retrieved back into the orbit (Figure 5). A titanium plate was fixed to repair the floor fracture. Post-operative fundus evaluation revealed a hyperemic optic disc with foveal blanching due to likely cilioretinal artery occlusion and multiple hemorrhages all over the retina (Figure 6). Although the optic nerve was intact, there was no response to any stimuli on visual evoked potential suggestive of traumatic optic neuropathy. However, the patient had an acceptable cosmetic appearance at 12 months.

## 3. Results

In all three cases, we were able to successfully and safely retrieve the intussuscepted globe with the described technique, reposition it within the orbital socket, and reconstruct the orbital floor and medial wall defect using a titanium mesh without globe rupture. In the third case, due to nearly complete displacement of the globe into the maxillary sinus, globe retrieval was assisted by Caldwell-Luc approach to provide better access.

## 4. Discussion

The term “intussusception” has not previously been applied to traumatic globe displacement and is formally proposed here to describe a distinctive morphological configuration in which the globe telescopes through an invaginated, swollen conjunctival-Tenon’s envelope, producing an apparent “empty-socket”. The word “intussusception” is derived from Latin “intus,” meaning “within” or “inside,” and “suscipere” meaning “to undertake.” These three cases differ from previously reported presentations as, despite meticulous eyelid retraction the globe remained clinically occult owing to its retraction through an invaginated, swollen conjunctival–Tenon’s sleeve. Cross-sectional imaging confirmed globe prolapse into the paranasal sinus, and intra-operative management required a controlled 360° conjunctival peritomy with suture-tagging of the recti to permit safe retrieval through a telescoped conjunctiva.

The transconjunctival approach has been specified in one case report earlier, but it did not involve a 360° peritomy and hooking of muscles [3]. Two of the three cases in our series suffered globe intussusception into the sinus following a bull horn injury. Bull horn injury can have varied presentations, from eyelid tear to globe rupture [4], and there has been only one case report with a bull horn injury leading to the globe in the maxillary sinus [5].

A review of the English-language literature from the years 1971 to 2024 was performed using the search terms: traumatic globe dislocation, maxillary sinus, ethmoid sinus and bull horn. Studies that included clinical and radiological diagnoses were included in our review. The data from previous studies is included in Table 1.

Thirty-five published case reports of traumatic dislocation of the globe into the sinuses were identified in the literature (Table 2). The age of these patients ranged from 10 to 68 years of age, and there was a clear male preponderance. More than 50% of these cases had suffered traffic accidents. Two of three cases in our series had a bull horn injury. Amongst the paranasal sinuses, traumatic globe dislocation mostly involved the maxillary sinus, but ethmoid sinus involvement has also been reported. Out of the five cases with globe luxation into the ethmoid sinus, four did not undergo any reconstruction of the medial wall, while one had a delayed reconstruction with a porous polyethylene implant.

The mechanism of traumatic globe dislocation is the same as that of orbital blow-out fracture along with variable soft tissue damage. Berkowitz et al. explained that the globe is supported within the orbit by the arrangement of the extraocular muscles, bulbar fascia, ligaments, and orbital fat [8]. It is known that even after removal of the maxilla and orbital floor, the globe does not completely sink into the maxillary sinus. A trauma forceful enough to disrupt these structures can cause globe dislocation.

It was hypothesized by Kreiner et al. that delay in treatment compromises blood supply to the optic nerve and increases the probability of irreversible vision loss due to twisting and stretching of the central retinal artery [20]. Our series could be different from the other singular case reports, where the conjunctiva had completely infolded to hide the globe. This telescoping of the conjunctiva is similar to intussusception, where a part of the intestine slides into the adjacent part. Including our three cases, we found that only 39.47% (15 out of 38) cases could recover any functional vision. Ten of these cases had been presented and surgically repositioned on the same day of trauma, one on the day after trauma, one after four days of trauma, while for three cases the interval between trauma and repair has not been documented.

Surgical management of globe dislocation is not standardized. Due to their presentation, such cases can be misdiagnosed as anophthalmic socket or auto-evisceration, and hence urgent imaging and immediate intervention are vital. Every attempt must be made to reposition the globe followed by orbital floor reconstruction. Repositioning of the globe has been reported by two techniques: (1) Direct traction by subconjunctival/sub-ciliary approach (2) Transmaxillary approach. We believe that the choice of approach should be guided by the proportion of dislocation and the intraoperative recognition of globe intussusception through the conjunctiva, an observation not previously described in the literature.

Whereas most orbital fractures are repaired within two weeks to optimize surgical planning, those with globe dislocation demand an urgent intervention with immediate reconstruction, often on the day of the injury, to restore ocular integrity and function. The urgency in such cases obviates the feasibility of patient-specific or three-dimensional printed implants [36], necessitating the use of immediately available standard materials. Various materials have been described in the literature for orbital wall reconstruction, including: titanium mesh, autogenous bone, porous polyethylene, and silicone [37].

Application of the PICO framework allowed a structured synthesis of the available literature on this rare but severe presentation of orbital trauma.

Population (P):

Across 35 published reports (and the current three cases), the affected cohort comprised predominantly young to middle-aged males, with a clear predominance of high-energy trauma, notably road traffic accidents (50%) and bull horn injuries. The maxillary sinus was the most commonly involved site (86%), followed by the ethmoid sinus. The rarity of this phenomenon emphasizes the need for heightened clinical suspicion in apparent “empty socket” presentations following periorbital trauma.

Intervention (I):

The principal intervention across studies was surgical retrieval and repositioning of the displaced globe, often accompanied by orbital wall reconstruction. Importantly, prior reports have not described the precise mechanism of dislocation into adjacent paranasal cavities; the clinical and intra-operative findings presented here provide the first detailed observations that help clarify likely pathways of displacement and inform choice of surgical approach. Our cases highlight a unique morphological variant, globe intussusception through the conjunctiva, necessitating a 360° peritomy and muscle tagging before repositioning. The addition of the Caldwell-Luc approach in one case facilitated safe retrieval from the maxillary sinus.

Comparison (C):

Reported approaches varied according to the extent and direction of displacement. Direct traction was used in partial dislocations, while transmaxillary and combined intraoral or subciliary routes were reserved for complete displacements. Functional visual recovery was higher when surgery was performed within 24 h of trauma. The transconjunctival approach with complete peritomy, as described in this series, offers controlled access with minimal additional trauma, though evidence from literature remains limited to anecdotal reports.

Outcome (O):

Among 38 collated cases (including the current three cases), only 39% achieved functional visual recovery. Early surgical intervention was the most consistent predictor of favorable outcomes. In our series, although anatomical repositioning was successful in all cases, visual recovery remained poor due to concurrent optic nerve or retinal injury. This emphasizes the need for rapid diagnosis, imaging, and urgent surgical intervention to preserve ocular integrity and optimize visual prognosis.

By integrating findings under the PICO framework, this review underlines that:

- Early recognition and imaging are critical to differentiate true enucleation from concealed intussusception.
- Prompt surgical retrieval, preferably within 24 h, improves anatomical and functional outcomes.
- Choice of approach should be tailored to the type of displacement, with conjunctival intussusception warranting a full peritomy and extraocular muscle tagging.
- Urgent reconstruction with available materials (titanium, porous polyethylene, or autogenous bone) is essential to restore orbital architecture.

Finally, this PICO-based synthesis offers a clearer evidence framework for future multicentric registries and outcome-based studies, with the goal of refining surgical strategies and optimizing both cosmetic and visual outcomes in traumatic globe dislocation.

In conclusion, three additional cases of traumatic globe luxation into the paranasal sinuses are reported and the term “globe intussusception” is formally introduced to describe the distinctive telescoping of the globe through the conjunctiva–Tenons envelope that can give the appearance of an anophthalmic socket. Clinical implications include: (1) assessment of orbital fractures by cross-sectional imaging and evaluation of the route of globe displacement are essential; (2) intra-operative recognition of conjunctival intussusception mandates a controlled 360° conjunctival peritomy with suture-tagging, cryoprobe or a bimanual technique using Caldwell–Luc access to permit safe retrieval and repositioning of the globe; and (3) urgent repositioning and timely orbital reconstruction are advised because functional visual recovery is limited; pooled data indicate that approximately 39% of patients regain useful vision. To standardize reporting and guide management, a pragmatic clinicoradiological classification is suggested for acceptance and future validation: Type I: partial displacement (globe displaced and enophthalmic but is visualized within the orbit); Type II: globe intussusception (conjunctival telescoping with an “empty socket” clinical appearance). Establishment of a prospective, multicenter registry and outcome studies is recommended to refine timing of intervention, optimize reconstructive strategies, and improve prognostic assessment of visual and cosmetic outcomes for this rare but surgically challenging injury.

## Figures and Tables

**Table 1:** Previously published cases of traumatic globe luxation (n = 35), into the maxillary and ethmoid sinus, arranged chronologically with authorship and year of publication, in English-language literature indexed (PUBMED) from 1971 to 2024.

Sr No.	Authors	Age	Sex	Etiology of Trauma	Involvement of Sinus	Time to Presentation	Globe Repositioning	Orbital Reconstruction	Visual Acuity
1	Emery et al., 1971 [6]	NA	NA	NA	Maxillary Sinus	NA	Direct traction	NA	NA
2	Emery et al., 1971 [6]	NA	NA	NA	Maxillary Sinus	Same day	Direct traction	NA	NA
3	Stasior, 1976 [7]	NA	M	Fire hose nozzle injury	Right Maxillary Sinus	NA	Trans-maxillary manual repositioning	No	Recovery 20/60
4	Berkowitz et al., 1981 [8]	24	F	Punch with fist	Left Maxillary Sinus	Same day	Trans-maxillary manual repositioning	Silicone implant	Recovery 20/20
5	Risco et al., 1984 [9]	40	M	Blunt trauma (hit with a wood block)	Left ethmoid sinus	5 days	Direct traction- trans-nasal repositioning	No	Light perception: recurrence of globe displacement
6	Singh et al., 1986 [5]	32	M	Bull horn		NA	Direct traction	No	6/36
7	Smit et al., 1990 [10]	21	F	Windshield injury	Right Maxillary Sinus	5 years	NA	Teflon plate	No
8	Ziccardi et al., 1993 [11]	25	M	Traffic accident	Left Maxillary Sinus	Same day	Sub-ciliary + Trans-maxillary	Autologous bone	20/50
9	Moon et al., 1997 [12]	40	M	Blunt instrument injury	Right Ethmoid Sinus	2 days	Direct Traction	No	No
10	Pelton et al., 1998 [13]	19	M	Traffic accident	Left Maxillary Sinus	Same day	Trans-maxillary manual repositioning	NA	NA
11	Tung et al., 1998 [14]	17	M	Traffic accident	Right Maxillary Sinus	-	Direct repositioning-sub-ciliary approach	Autogenous bone graft	No
12	Saleh & Leatherbarrow, 1999 [15]	29	M	Blunt trauma	Left Maxillary Sinus	2 Months	Enucleation	No	No
13	Tranfa et al., 2000 [16]	58	M	Fall of a tree branch	Left Ethmoid Sinus	Same day	Direct Traction	No	Recovery 20/100
14	Kim and Baek, 2005 [3]	68	M	Traffic accident	Right Maxillary Sinus	Same day	Direct traction- transconjunctival	Porous polyethylene	No
15	Okabe et al., 2005 [17]	37	M	Injury with a piece of wood	Left Ethmoid Sinus	Same day	Direct Traction	No reconstruction of medial orbital wall	Recovery 20/15 restricted motility
16	Müller-Richter et al., 2007 [18]	62	M	Blunt trauma by machine	Right Maxillary Sinus	Same day	Trans-maxillary manual repositioning	Balloon catheter left inside the sinus	Recovery 20/20
17	Abrishami et al., 2007 [19]	18	M	Traffic accident	Right Maxillary Sinus	7 days	Direct repositioning sub-ciliary approach	Porous polyethylene	No
18	Kreiner et al., 2008 [20]	50	M	Wall collapse injury	Left Maxillary Sinus	Same day	Direct traction	Silicone sheet	No
19	Ramstead et al., 2008 [21]	32	M	Stepped by a bull	Left Maxillary Sinus	Same day	NA	Titanium mesh	Recovery 20/200
20	Jellab et al., 2008 [22]	24	M	Traffic accident	Left Maxillary Sinus	Same day	NA	Autogenous bone	Light perception
21	Jellab et al., 2008 [22]	50	F	Traffic accident	Left Maxillary Sinus	Same day	No repositioning	No	NA
22	Akhaddar et al., 2010 [23]	62	M	Traffic accident	Right Maxillary Sinus	NA	NA	NA	NA
23	Gulati et al., 2011 [24]	10	M	Bicycle accident	Right Maxillary Sinus	NA	Trans-maxillary	Balloon Catheter inside sinus	Recovered 6/16
24	Zhang-Nunes et al., 2012 [25]	20	M	Traffic accident	Right Maxillary Sinus	Same day	NA	Sialistic sheet	Recovery 20/25
25	Xu et al., 2013 [26]	46	F	Traffic accident	Right Maxillary Sinus	NA	Direct reposition	Titanium mesh	Light perception
26	Haggerty and Roman, 2013 [2]	35	M	Traffic accident	Left Maxillary Sinus	Same day	Trans-maxillary manual repositioning	Titanium mesh	Light perception
27	Amaral and Nery, 2015 [27]	44	M	Struck by gym weight	Left Maxillary Sinus	4 days	Trans-maxillary manual repositioning	Titanium mesh	Recovery 20/50
28	Wang JM et al., 2017 [28]	15	M	Accident riding ATV	Left Maxillary Sinus	NA	NA	Titanium mesh	No
29	Noman SA and Shindy MI, 2017 [29]	24	F	Blunt wooden object	Right Maxillary Sinus	Same day	Trans-maxillary approach	Titanium Mesh	Recover 20/40
30	Alam et al., 2017 [30]	45	F	Trauma with door	Right Maxillary Sinus	3 days	Direct repositioning	Titanium mesh	VA: counting fingers to 1 m
31	Bastos et al., 2021 [31]	27	M	Punch in the eye	Right Maxillary Sinus	Same day	Trans-maxillary approach	14 days after repositioning of globe, reconstruction with titanium	No VA
32	Onaran et al., 2021 [32]	36	F	Blunt trauma on door handle	Left Ethmoid Sinus	Same day	Gentle intranasal digital manipulation	4 months after repositioning, with porous polyethylene implant	VA 20/20
33	Gobeaut et al., 2022 [33]	54	F	Fall from height	Right Maxillary Sinus	1 day	Direct traction and trans-maxillary approach	Resorbable polydiaxone osteosynthesis implant (PDS)	VA 20/125
34	Steel DA et al., 2023 [34]	-	M	Traffic Accident	Left Maxillary Sinus	Same day	Trans-maxillary	Titanium mesh	No perception of light
35	Vergez P et al., 2024 [35]	67	M	Fall from a height	Left Maxillary Sinus	Same day	Direct Traction	Titanium mesh	20/50

**Table 2:** Clinical summary of 35 cases of globe intussusception into the maxillary and ethmoid sinus published in English-language literature indexed (PUBMED) from 1971 to 2024.

Total Number	Age Range	Gender	Etiology of Trauma	Involvement of Sinus	Time to Presentation	Globe Repositioning	Orbital Reconstruction	Visual Acuity
35	10–68 Years	Male: 25 Female: 8 Not mentioned: 2	Traffic accident: 14 Others: 9 NA: 2	Maxillary Sinus: 30 Ethmoid Sinus: 5	Range: Same day to 5 years	Direct reposition: 15 Transmaxillary: 12 NA: 6 No reposition: 1 Enucleation: 1	Titanium: 9 Silicone: 3 Porous poly-ethylene: 3 PDS: 1 Bone: 3 No reconstruction: 9 Others: 3, NA: 4	Functional Vision improved: 15 Finger counting 1 m: 1 Light perception: 4 Not improved: 10 NA: 5

## Data Availability

The original contributions presented in this study are included in the article. Further enquiries can be directed to the corresponding author.

## Abbreviations

The following abbreviations are used in this manuscript:

ATV	All-Terrain Vehicle
M	Male
F	Female
NA	Not Applicable
